# Potentiometric MIP-Modified Screen-Printed Cell for Phenoxy Herbicides Detection

**DOI:** 10.3390/ijerph192416488

**Published:** 2022-12-08

**Authors:** Camilla Zanoni, Stefano Spina, Lisa Rita Magnaghi, Marta Guembe-Garcia, Raffaela Biesuz, Giancarla Alberti

**Affiliations:** 1 Department of Chemistry, University of Pavia, Via Taramelli 12, 27100 Pavia, Italy; 2 Unità di Ricerca di Pavia, INSTM, Via G. Giusti 9, 50121 Firenze, Italy; 3 Departamento de Química, Facultad de Ciencias, Universidad de Burgos, Plaza de Misael Bañuelos s/n, 09001 Burgos, Spain

**Keywords:** 2-methyl-4-chlorophenoxyacetic acid (MCPA), herbicides, screen-printed electrodes, molecularly imprinted polymers (MIP), MIP-based electrodes, potentiometric screen-printed cell, electroanalysis, analytical chemistry

## Abstract

In this study, a molecularly imprinted polymer (MIP)-based screen-printed cell is developed for detecting phenoxy herbicides using 2-methyl-4-chlorophenoxyacetic acid (MCPA) as the template. MCPA is a phenoxy herbicide widely used since 1945 to control broadleaf weeds via growth regulation, primarily in pasture and cereal crops. The potentiometric cell consists of a silver/silver chloride pseudo-reference electrode and a graphite working electrode coated with a MIP film. The polymeric layer is thermally formed after drop-coating of a pre-polymeric mixture composed of the reagents at the following molar ratio: 1 MCPA: 15 MAA (methacrylic acid): 7 EGDMA (ethylene glycol dimethacrylate). After template removal, the recognition cavities function as the ionophore of a classical ion selective electrode (ISE) membrane. The detected ion is the deprotonated MCPA specie, negatively charged, so the measurements were performed in phosphate buffer at pH 5.5. A linear decrease of the potential with MCPA concentration, ranging from 4 × 10^−8^ to 1 × 10^−6^ mol L^−1^, was obtained. The detection limit and the limit of quantification were, respectively, 10 nmol L^−1^ and 40 nmol L^−1^. A Nernstian slope of about −59 mV/dec was achieved. The method has precision and LOD required for MCPA determination in contaminated environmental samples.

## 1. Introduction

In the last few years, the use of pesticides has grown due to the demand of the market that claims more and more abundant and leafy products. The first synthetic herbicides were produced during the Second World War to remove crop weeds. The use of herbicides can be helpful for the agricultural field, but they may degrade and contaminate the environment. These pollutants can cause human diseases, so detecting and quantifying them is crucial [1].

MCPA (2-methyl-4-chlorophenoxyacetic acid) was the first synthetic herbicide synthesized in England around 1941. It is a selective phenoxy herbicide that controls broadleaf weeds in arable and cereal crops, acting on growth hormones. MCPA can mimic auxin, encouraging uncontrolled growth and subsequent death of certain plants, such as dicotyledons. It is synthesized by exposing 4-chloro-2-methylphenol to chloroacetic acid in diluted alkaline solutions [2]. MCPA has a soil half-life of 24 days and a water-octanol partition coefficient of 0.2–1 mL/g, but some environmental pH and temperature values may facilitate the sorption and the degradation [3]. Interestingly but alarmingly, MCPA and similar phenoxy herbicides can form stable complexes with toxic metal ions, such as lead and cadmium, in environmental pH ranges, increasing their bioavailability; for this reason, it is fundamental to detect these pesticides in the environment [4].

As far as toxicity is concerned, these herbicides, and in particular the most common MCPA, are approved for use in all EU countries (except Cyprus), the United States, China, Canada, Australia, and New Zealand [5] since their toxicity depends on the species affected and the method of exposure to the pesticide. In fact, for example, MCPA has a very low bioconcentration factor (BCF) 1–14 [6], which means that the bioaccumulation rate is low, and if excessive exposure to the poison kills an organism, it is not dangerous for other organisms [7]. The human symptoms due to MCPA poisoning are fatigue, weakness, anoxia, nausea, vomiting, diarrhea, lowering of blood pressure, body temperature disturbance, progressive hypotension, ataxia, neuromuscular inability, and convulsion [8], and the human lethal oral concentration is around 814 mg/Kg [9].

Moving to phenoxy herbicides detection, the literature reports many chromatographic [10,11,12,13,14] and voltammetric [15,16,17,18,19] methods, but no potentiometric ones are described. Potentiometric and, more in general, all electrochemical techniques have several advantages compared to the classic ones: the instrumentation is less expensive, the time of analysis is low, the apparatus may be portable, the sensors can be used for in situ measurements, and the surface of the electrodes can be modified in order to obtain more specific and sensitive detections. In particular, the electrodic surface can be activated with different kinds of modifiers such as multi-Walled carbon nano tubes (MWCNTs) [20,21,22,23,24,25], metal nano particles (NPs) [26,27,28,29,30,31], graphene [32,33,34,35,36,37], metal organics frameworks (MOFs) [38,39,40,41,42,43] in order to enhance the sensitivity, aptamers, antibodies [44,45,46,47,48,49], enzymes [50,51,52,53,54,55], or molecularly imprinted polymers (MIPs) [56,57,58,59,60,61] to improve the selectivity.

**Figure 1:**
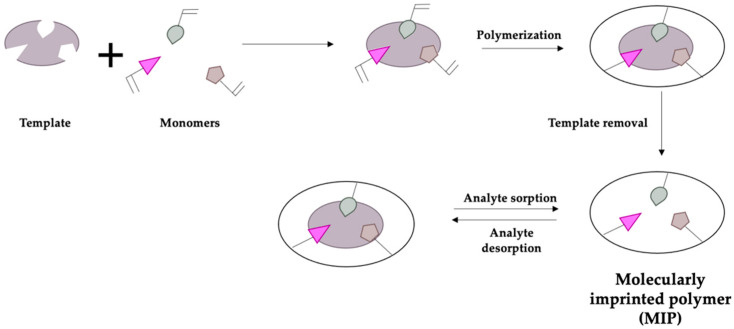
Schematic representation of molecularly imprinting procedure to form a MIP.

Among these receptors for electrode surface modification, MIPs represent an emerging, interesting topic in polymeric chemistry that has recently caught on since these polymers can be used as synthetic receptors, replacing biomolecules such as enzymes, aptamers, and others [62]. MIPs are characterized by the presence of selective cavities meant to sorb a target molecule; specific monomers must be chosen to obtain these cavities since they have to interact through weak bonds with a template molecule, i.e., the specific analyte. Once the polymerization is completed, the template molecule must be removed to obtain a selective cavity with the shape of the analyte [63]. Figure 1 reports the schematic procedure to obtain a molecularly imprinted polymer:

MIPs offer several advantages: they can be designed selectively for the target molecule using different monomers, the monomers used are usually low-cost, reusable, and easily storable, and conductive polymers can also be obtained [64]. Conversely, enzymes and other biological receptors are costly, must be stored in certain conditions, and, generally, biosensors have a short lifetime [65].

**Figure 2:**
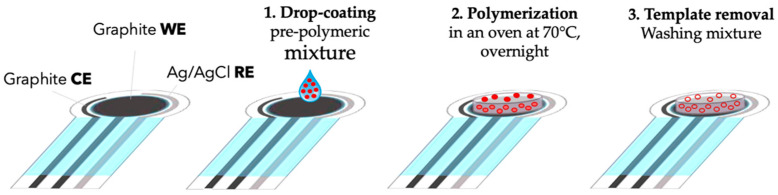
Schematic representation of MIP-modified screen-printed cell assembly.

In this context, the present study aims to develop a MIP-based screen-printed potentiometric cell to detect phenoxy herbicides using MCPA as a template molecule. The sensor is prepared according to a previously developed procedure [56] schematized in Figure 2. The MIP-modified working electrode acts as an ion-selective electrode, so the solution pH required for the measurements has to be selected in a range in which MCPA is negatively charged. The pH value chosen is 5.5 according to the MCPA p*K*a of 3.07 [66]. The method is tested on synthetic solutions and applied to fortified natural water samples.

## 2. Materials and Methods

### 2.1. Reagents and Materials

Methacrylic acid (MAA) (Sigma-Aldrich, Milan, Italy) and ethylene glycol dimethacrylate (EGDMA) (Sigma-Aldrich) were filtered by using a column of aluminum oxide (Sigma-Aldrich) in order to remove stabilizers. 2,2-azobisisobutyronitrile (AIBN), MCPA, Mecoprop, Dichloroprop, 2,4-D Pestanal, acetic acid, and ethanol were used as received from Sigma-Aldrich (Milan, Italy). Phosphate buffer solutions (PBS buffer) were prepared in ultrapure water, adjusting the pH with hydrochloric acid or sodium hydroxide (Sigma-Aldrich, Milan, Italy). Solutions for electrode surface characterization were prepared from sodium chloride, potassium chloride, and potassium hexacyanoferrate(III) (Sigma-Aldrich, Milan, Italy). Tap water samples were obtained from the lab sink (Department of Chemistry, University of Pavia). Three-electrodes screen-printed cells with a graphite-ink working electrode, a graphite-ink counter electrode, and Ag/AgCl-ink pseudo-reference electrode were obtained by Topflight Italia SPA (Vidigulfo, Pavia, Italy).

### 2.2. Instruments

The ultrasonic bath AU-32 Argo Lab (Tecno-Lab, Milan, Italy), with ultrasound power 120 W, was employed for the pre-polymeric mixture preparation.

pH measurements were performed with a pH-meter Mettler Toledo mod. SevenMulti, equipped with a combined glass electrode InLab Pro (Mettler Toledo, Milan, Italy).

Potentiometric analyses and impedance spectroscopy measures were performed with the potentiostat/galvanostat EmStat4s-PalmSens BV (Houten-The Netherlands https://www.palmsens.com/product/emstat4s/ (accessed on 7 June 2022)).

Scanning electron microscopy (SEM) images were acquired with EVO MA10 scanning electron microscope. The measurements were performed under an ultra-high vacuum with an electron generation voltage of 20 kV and a working distance of 8.5 mm.

### 2.3. MIPs Pre-Polymeric Mixture

The pre-polymeric mixture was prepared by dissolving 28 mg of MCPA (0.139 mmol) with 175 μL of MAA (2.07 mmol), 175 μL of EGDMA (0.927 mmol), AIBN 35 mg, and 200 μL of toluene. The so obtained pre-polymeric mixture (with a molar ratio of 1:15:7 = MCPA:MAA:EGDMA) was sonicated for 30 min and then deaerated for 3 min with N_2_.

The pre-polymeric mixture for the non-imprinted polymer (NIP) was prepared following the same procedure but without adding the template MCPA.

### 2.4. Working Electrode Modification

Each screen-printed cell (SPC) was washed with ethanol and dried with N_2_. Then, 7 μL of pre-polymeric mixture was drop-coated on the working electrode (WE) surface. The SPC was then placed in an oven at 70 °C for 12 h for thermo-induced polymerization. After polymerization, the SPC was washed, immersing the SPC in 10 mL of a mixture of ethanol:acetic acid = 4:1 under gentle stirring. Five cycles of 30 min each to remove the template and the unreacted monomers as checked by analyzing the UV–vis spectrum of the mother waters at each washing step. Before use, the sensor was placed in ultrapure water for 10 min.

Screen-printed electrodes are disposable, so they do not require particular maintenance procedures. After modification with the MIP/NIP layer, no more than two experiments (calibration or sample analysis) can be performed with the same electrode without compromising the measurements.

### 2.5. Electrochemical Impedance Spectroscopy (EIS)

For the electrodic surface characterization, electrochemical impedance spectroscopy (EIS) measurements were performed using the potentiostat/galvanostat EmStat4s (EmStat4s-PalmSens BV (Houten-The Netherlands), using as electrochemical probe a solution of 5 mmol L^−1^ K_4_Fe(CN)_6_/0.1 mol L^−1^ KCl at pH 7.2 and the following instrumental parameters: frequency range from 100 kHz to 10 mHz and signal amplitude of 50 mV. EIS measurements were performed on the washed MIP-modified electrodes, NIP-modified electrodesm and the MIP-modified electrode after immersion in 10^−7^ mol L^−1^ MCPA solution.

### 2.6. Potentiometric Measurements

Potentiometric measurements were performed by dipping the washed MIP-modified screen-printed cell in 15 mL of PBS buffer at pH 5.5 and adding portions of 20 μL of MCPA standard solution 25 nmol L^−1^ under gentle stirring. At each addition of MCPA, the potential was recorded, and the average of the values sampled in the last 60 s after waiting 3 min for the steady-state was considered the response signal (stability criterium ΔE/Δt = 0.02 mV/s, see Figure S1 in Supplementary Materials). Figure S2 shows the experimental setup (see Supplementary Materials).

## 3. Results

### 3.1. Working Electrode Modification by a MIP Layer and Characterization Using EIS

The surface of the working electrode was functionalized by a MIP layer to obtain a selective sensor. The pre-polymeric mixture molar ratio was defined according to previous studies [56]. The volume selected for drop-coating was 7 µL, i.e., a quantity enough to cover the working electrode and keep the pseudo-reference and the counter electrodes unaltered. MAA was chosen as the functional monomer to obtain the best imprinting features since its carboxylic groups can interact with the template molecule through hydrogen bonds. EGDMA was employed as the crosslinker, toluene as the porogenic solvent, and AIBN as the radical initiator. The formation of the MIP cavities was verified by SEM measurement on MIP and NIP powders obtained by polymerization in bulk (see Supplementary Materials, Figure S3). For the MIP material, filamentous and compact particles of 10/20 microns appeared; conversely, only larger and uniform particles can be observed for NIP.

Electrochemical impedance spectroscopy (EIS) is a useful technique for characterizing the surface of a modified electrode [67]; indeed, EIS can help to understand the changing of the conducting properties of a modified electrodic surface, schematizing the phenomena that are taking place at the interface electrode/electrolyte. Moreover, the results of EIS measurements can be helpful as a check of the successful formation of the imprinting polymer film. The data obtained by the electrochemical impedance spectroscopy can be summarized in the Nyquist plot, and they may be schematized using a Randles equivalent circuit [68].

**Figure 3:**
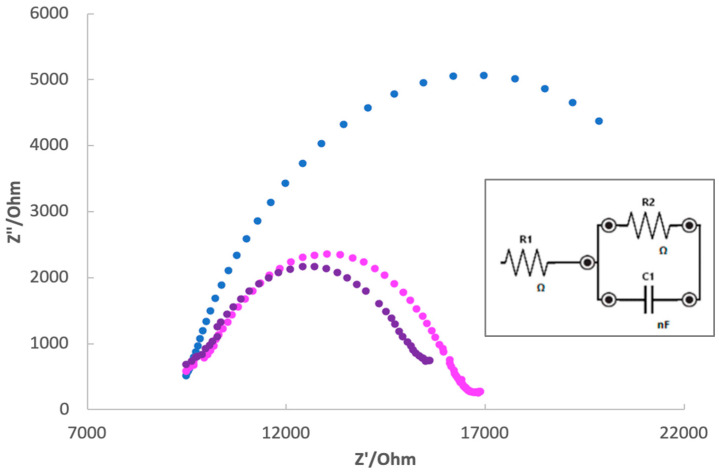
Nyquist plot of electrode modified with NIP (blue), washed MIP (purple) and MIP re-charged with MCPA 10^−7^ mol L^−1^ (pink). Measurements were performed in 5 mmol L^−1^ K_4_Fe(CN)_6_/0.1 mol L^−1^ KCl at pH 7.2. The insert in the figure is the Randles equivalent circuit of the trends reported in the Nyquist plot for all three modified electrodes.

For the present study, the measurements were performed using 5 mmol L^−1^ K_4_Fe(CN)_6_/0.1 mol L^−1^ KCl at pH 7.2 as the electrochemical probe and supporting electrolyte. The Nyquist plot (Figure 3) of the modified electrodes by respectively NIP, re-charged MIP (after equilibration with 10^−7^ mol L^−1^ MCPA solution), and washed MIP showed a semicircle; this is a typical pattern of the surface-modified electrodes when the electron transfer is the limiting step. It can be observed that the resistance to the electron transfer at the electrode/electrolyte interface increases passing from the washed MIP to the NIP; this behavior means that the non-imprinted polymer hinders the flow of charges to the electrode surface more than the washed imprinted polymer. This assumption is confirmed by the impedance behavior of the not-washed MIP, which showed the highest resistance value; probably, the cavities occupied by the template prevented the flow of charges through the membrane. The diameter of the semicircle reported in the Nyquist plot corresponds to the resistance of the electrode, confirming the non-conductive nature of the polymer used to modify the working electrode surface.

The results represented by the Nyquist plot were schematized using an equivalent circuit helpful to separate any contribution to the total system impedance (Randles equivalent circuit, insert in Figure 3). The equivalent circuit used for fitting the experimental points is characterized by the presence of R_1,_ which corresponds to the solution resistance; R_2,_ which corresponds to the resistance of the electrode/electrolyte interface; and C1, which corresponds to the double layer capacitance.

### 3.2. Potentiometric Measurements by MIP-Modified Screen-Printed Cell

**Figure 4:**
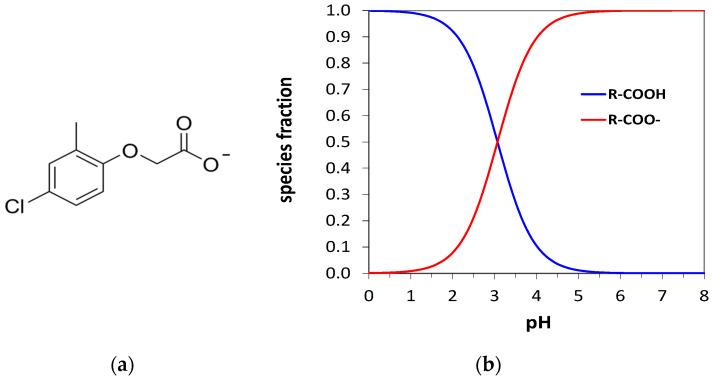
(**a**) Negatively charged deprotonated form of MCPA. (**b**) Graph of species distribution in function of the pH for MCPA.

After the screen-printed cell modification with the MIP layer and after the removal of the template by the washing procedure previously described, it can be used to perform potentiometric measurements. For obtaining a potentiometric response, the analyte must be charged. For this reason, given that the MCPA p*K*a = 3.07, measurements were performed in PBS buffer pH 5.5 to ensure that the predominant species at this pH is the negatively charged deprotonated form [66]. Figure 4a shows the anion structure, and Figure 4b the graph of species distribution in function of the pH for MCPA.

**Figure 5:**
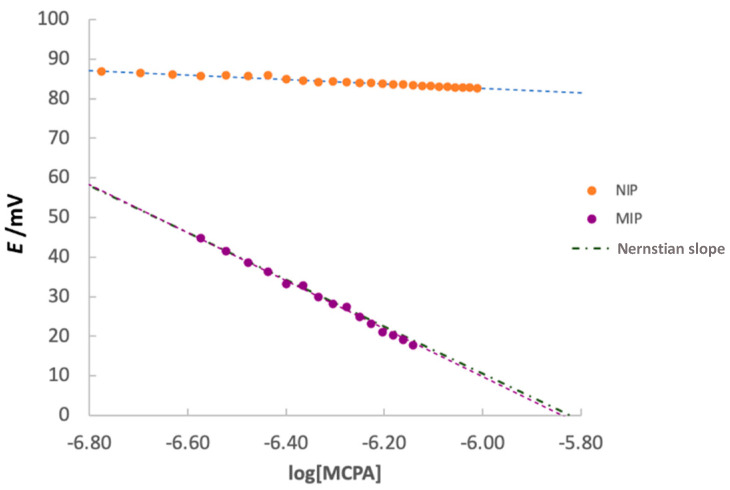
Calibration curve of MCPA of screen-printed cell modified with MIP (purple), with NIP (orange), and theoretical Nernstian slope. Experimental conditions: 15 mL PBS pH 5.5, additions of 0.02 mL MCPA std 25 µmol L^−1^.

Potentiometric measurements were performed by dipping the modified screen-printed cell in 15 mL of PBS buffer at pH 5.5 for 3 min under gentle stirring after reaching the potential stability (stability criterium ΔE/Δ*t* = 0.02 mV/min). The calibration curve was obtained by adding portions of 20 µL of MCPA standard 25 µmol L^−1^ to the buffer and plotting the potential (*E*, mV) vs. the logarithm of MCPA concentration (log[MCPA]). Figure 5 shows an example of the calibration curves obtained for MIP and NIP functionalized electrodes.

**Figure 6:**
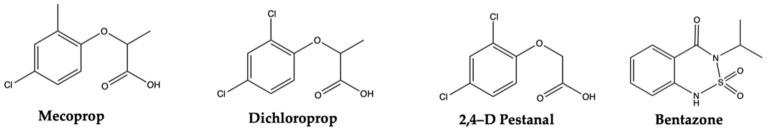
Molecular structure of interferents.

Calibrations were performed with three different MIP or NIP-modified electrodes. Each electrode was used twice, so the standard deviation is calculated on the slopes obtained from 6 different calibrations using the three different electrodes two times each. As an example, one of these calibrations is shown in Figure 6. The slope of the calibration plots (expressed as the average value of the six experiments) for the electrodes modified with MIP is −59(1) mV/dec, very similar to the theoretical −59.2 mV/dec, expected from the Nernst equation. Conversely, the slope of the NIP-modified electrodes is much lower (of about −9(11) mV/dec); this means that these electrodes are lightly sensitive to the presence of MCPA in solution due to the unspecific interactions between the analyte and the polymeric film.

The detection limit (LOD) and the quantification limit (LOQ) for the potentiometric measurements with the MIP-modified electrodes can be obtained from the following relations (Equations (1) and (2)):

(1) $$\mathrm{LOD}=\frac{3.3\cdot s_{y/x}}{\mathrm{slope}}$$

(2) $$\mathrm{LOQ}=\frac{10\cdot s_{y/x}}{\mathrm{slope}}\;$$

by considering the linearized Nernst equation 10^E/slope^ vs. MCPA (mol L^−1^). *s_y_*_/*x*_ is the standard deviation of y-residuals (i.e., the random errors in the y-direction); *s_y_*_/*x*_ can be considered not significantly different from the standard deviation of replicate measurements of blank solutions [69].

The LOD and the LOQ obtained are, respectively, 13 nmol L^−1^ and 40 nmol L^−1^, demonstrating high sensitivity, although it is a potentiometric method.

For comparison in Table 1, the detection limits for MCPA analysis obtained from the present and other electroanalytical methods are reported.

### 3.3. Interferences

In order to study the selectivity of the sensor, tests were performed, investigating the pesticides shown in Figure 6, three structurally analogues (Mecroprop, Dichloroprop and 2,4-D Pestanal) and one of a different structure of MCPA (Bentazone).

Table 2 summarizes the slope of the calibration curves (E/mV vs. log[pesticide]) obtained by analyzing the interferents without any MCPA.

As expected, the phenoxy-based molecules, similar to MCPA, show a slope very close to the theoretical one of −59.2 mV/dec, while the MIP-functionalized screen-printed cell is not sensitive to the presence of bentazone; indeed, in this case, the slope is about −1 mV/dec.

To corroborate these results, other measurements were conducted in solutions containing MCPA at a constant concentration (0.5 µmol L^−1^) and increasing quantities of each interferent. As foreseeable, the sensor is sensitive to the presence of both MCPA and another phenoxy herbicide when they are in solution together, while it is sensitive to only MCPA in solutions containing bentazone.

Table 3 reports the slope of the calibrations performed. For the phenoxy herbicides, in the *x*-axis of the calibration plots, the logarithm of the sum of both herbicides’ concentrations is reported; conversely, the logarithm of the MCPA concentration was considered for the calibration plot obtained in the presence of bentazone. For this last pesticide, it was seen that the sensor is sensitive only to the presence of MCPA. Moreover, as further confirmation, another experiment was performed by adding increasing quantities of bentazone (ranging from 0.5 mmol L^−1^ to 2.5 mmol L^−1^) to a solution containing MCPA 0.5 mmol L^−1^: In this experiment, the potential does not change after each addition of Bentazone (see Figure S4, Supplementary Materials).

It is interesting to highlight that the MIP-base electrode is selective only to phenoxy herbicides with similar structure and chemical behavior to the template MCPA so the sensor could be applied to determining the total degree of contamination from similar substances.

### 3.4. Real Samples Analyses

In order to verify the applicability of the MIP-based potentiometric screen-printed cell to phenoxy herbicides detection in environmental samples, some experiments were performed testing tap water samples spiked with MCPA; the quantification was performed by the standard addition technique. The tap water samples’ pH was adjusted to 5.5 and spiked with different concentrations of MCPA. The % recovery and the RDS were calculated and reported in Table 4.

The standard addition method is used to determine the concentration of MCPA in the spiked samples as the usual procedure for electrochemical analyses of real samples. The straight lines obtained during the measurements have slopes ranging from −57(3) mV/dec and −62(2) mV/dec, so the sample’s matrix does not affect the sensitivity and the LOD of the electrode

As can be observed in Table 4, a good % recovery, ranging from 98.5% and 103.6%, was obtained; the precision was quite good since the highest value of the % RDS is 4.6.

These results were promising for practical applications of the developed potentiometric sensor for environmental analyses of samples contaminated by phenoxy herbicides.

## 4. Conclusions

This paper presents a potentiometric screen-printed cell modified with MIP for phenoxy herbicides detection using MCPA as the template molecule. The molecularly imprinted polymer (MIP) film over the working electrode surface recognizes these herbicides thanks to the specific cavities interacting with the analyte through weak bonds. It acts like an ionophore of a classical ion-selective electrode when the target analyte is present as an anion in aqueous solutions.

Electrochemical impedance spectroscopy (EIS) was applied to characterize the surface of the modified working electrode, confirming the non-conductive nature of the acrylic polymer film coverage.

Calibration studies were performed, and the slope of the linear plot E/mV vs. log[MCPA] was Nernstian, being equal to −59 mV/dec.

The interferents tests highlighted similar sensing properties of the MIP-modified screen-printed electrode to MCPA for Mecoprop, Dichloroprop, and 2,4-D Pestanal, i.e., phenoxy pesticides structurally similar to the target one, confirming that the sensor could be applied to determine the total degree of contamination coming from similar substances. Conversely, the MIP-based electrode does not respond to molecules of very different structures, such as the Bentazone examined here.

The low detection limit (about 10 nM) and the good results obtained from recovery tests on spiked tap water samples are promising for future sensor applications. It can be highlighted that the sensors can directly detect phenoxy herbicides present in environmental waters at concentrations compared to the World Health Organization (WHO) guideline value (2 µg L^−1^ = 10 nmol L^−1^) [7]. It is not sensitive enough to quantify, without a preconcentration step, the herbicides at concentration levels required by the European Commission Drinking Water Directive 98/83/EC, concerning the quality of water for human consumption, i.e., 0.1 μg L^−1^ = 0.5 nmol L^−1^) [7].

## Figures and Tables

**Table 1:** Detection limits of different electroanalytical methods for MCPA analyses.

Electrode, Method	LOD (nmol L^−1^)	Reference
Glassy carbon, DPV ^1^	8	[19]
PANI-β-CD/fMWCNT/GCE, CV ^2^	990	[16]
Activated glassy carbon, SWV ^3^	1.6	[70]
PANI-β-CD/fMWCNT/GCE, CV ^2^	1100–1600	[15]
Silica-modified carbon paste electrode, DPV ^1^	1300	[18]
MIP-modified SPC ^4^, potentiometry	13	This work

^1^ DPV, differential pulsed voltammetry; ^2^ CV, cyclic voltammetry; ^3^ SWV, square wave voltammetry; ^4^ SPC, screen-printed cell.

**Table 2:** Slopes of the calibration curves for each pesticide (average values obtained for three calibration curves for each pesticide). Numbers in parentheses are the standard deviation on the last digit.

Pesticide	Slope (mV/dec)
Mecoprop	−54 (3)
Dichloroprop	−57 (5)
2,4-D Pestanal	−55 (3)
Bentazone	−1.3 (2)
**MCPA**	**−59 (1)**

**Table 3:** Slope of the calibration curves performed in solutions containing 0.5 µmol L^−1^ of MCPA and increasing quantities of each interferent. For the phenoxy herbicides, in the *x*-axis of the calibration plots, the logarithm of the sum of both herbicides’ concentrations is reported; conversely, the logarithm of the MCPA concentration was considered for the calibration plot obtained in the presence of bentazone.

Pesticide	Slope (mV/dec)
MCPA + Mecoprop	−57 (2)
MCPA + Dichloroprop	−55 (3)
MCPA + 2,4-D Pestanal	−57 (4)
MCPA+ Bentazone	−60 (2)
**MCPA**	**−59 (1)**

**Table 4:** Recovery experiments. Tap water samples adjusted to pH 5.5 with PBS and spiked with different MCPA concentrations.

Spike Concentration (μmol L^−1^)	Found (μmol L^−1^, *x* ± CI ^1^)	% RDS	% Recovery
0.169	0.166 ± 0.04	2.5	98.5
0.101	0.101 ± 0.05	4.6	103.6
0.531	0.5338 ± 0.02	0.5	100.5
0.918	0.928 ± 0.03	1.1	101.1

^1^ CI = 95% confidence interval. For 3 replicates, *t* = 4.30.

## Data Availability

Not applicable.

## Supplementary Materials

The following supporting information can be downloaded at: https://www.mdpi.com/article/10.3390/ijerph192416488/s1, Figure S1: Potentiometric response of a MIP-based screen-printed cell at different MCPA concentrations in PBS buffer at pH = 5.5. Steady-state reached in 3 min. Cell potential registered in the last 60 s. Figure S2: (a) Screen-printed xell; (b) Experimental setup: the electrode is dipped in the buffer solution and connected to (c) EmStat 4s for potential acquisition; Figure S3: SEM images of (a) MCPA molecularly imprinted polymer and (b) not-imprinted polymer at the same magnification; Figure S4: Potentiometric response of the MIP-modified electrode in MCPA 0.5 µmol L^−1^ solution after the addition of bentazone of 0.5 µmol L^−1^, 1.0 µmol L^−1^ and 2.5 µmol L^−1^.
