# Supplementary material for: Potentiometric MIP-Modified Screen-Printed Cell for Phenoxy Herbicides Detection
Source: Int J Environ Res Public Health. 2022 Dec 8;19(24):16488. doi: 10.3390/ijerph192416488 (PMC9779394; doi:10.3390/ijerph192416488)
Supplement: Supplementary file 1 [file ijerph-19-16488-s001.zip › ijerph-2023734-supplementary.pdf]

Supplementary materials

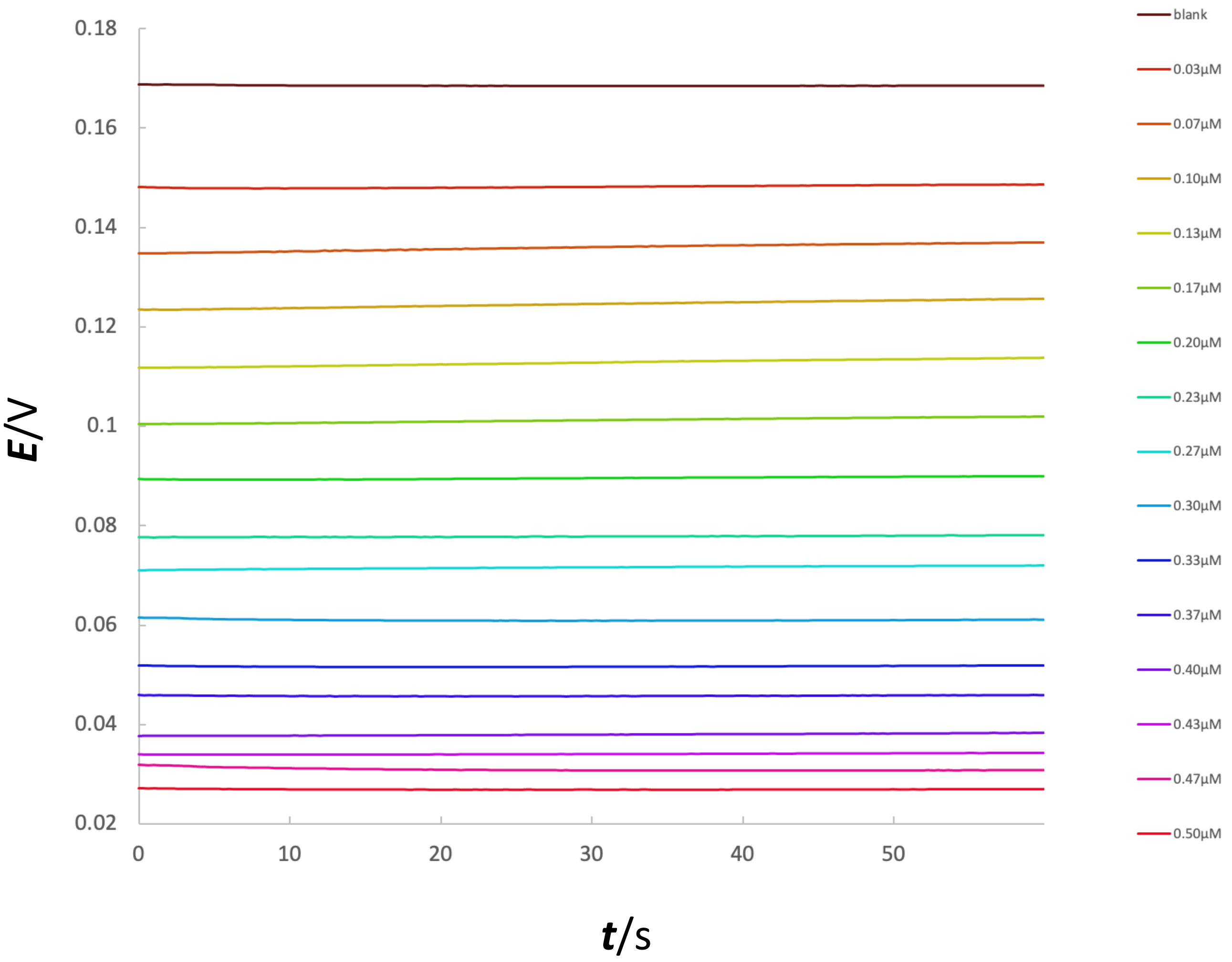

Figure S1. Potentiometric response of a MIP-based screen-printed cell at different MCPA concentrations in PBS buffer at pH = 5.5. Steady-state reached in 3 min. Cell potential registered in the last 60 s.

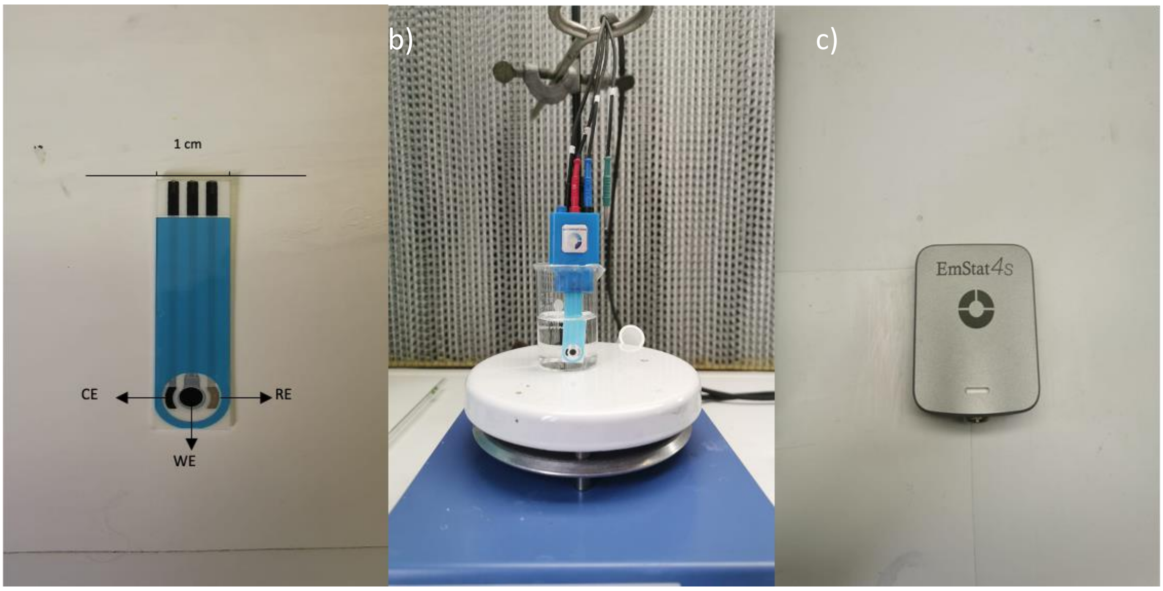

Figure S2. a) Screen Printed Cell; b) Experimental set-up: the electrode is dipped in the buffer solution and connected to c) EmStat 4s for potential acquisition.

a) b)

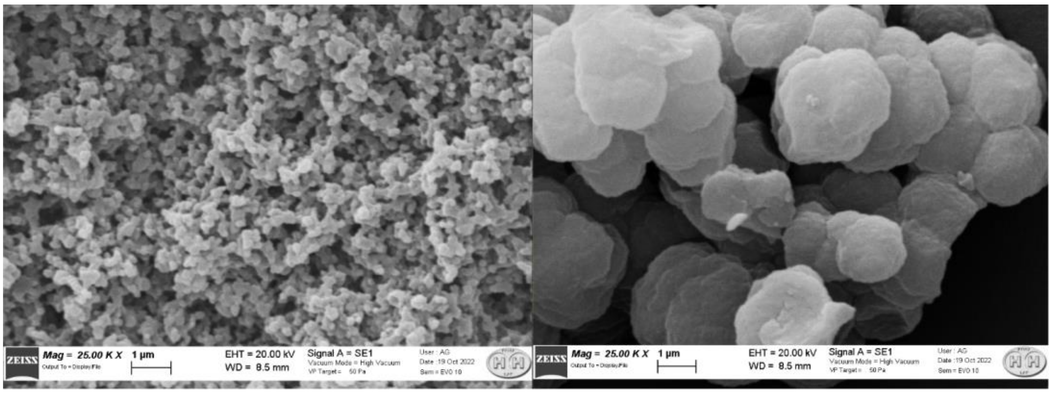

Figure S3. SEM images of (**a**) MCPA molecularly imprinted polymer and (**b**) not-imprinted polymer at the same magnification

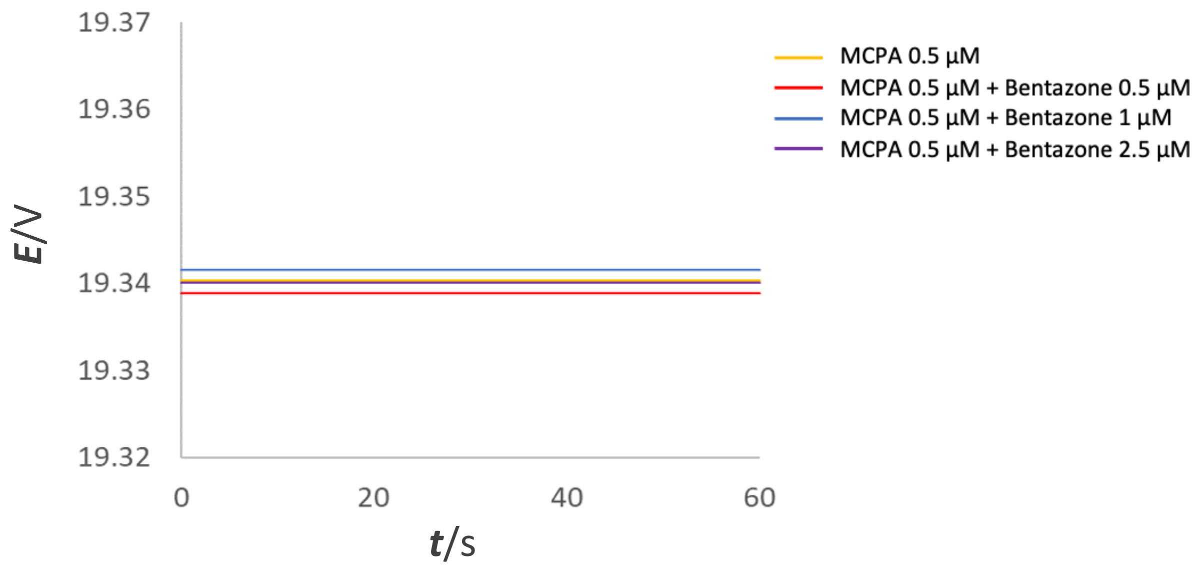

Figure S4. Potentiometric response of the MIP-modified electrode in MCPA 0.5 µmol L^{-1} solution after the addition of Bentazone of 0.5 µmol L^{-1}, 1.0 µmol L^{-1} and 2.5 µmol L^{-1}.
